# A race through the maze of genomic evidence

**DOI:** 10.1186/gb-2008-9-s1-s1

**Published:** 2008-06-27

**Authors:** Timothy R Hughes, Frederick P Roth

**Affiliations:** 1 Donnelly Centre for Cellular and Biomolecular Research and Banting and Best Department of Medical Research, University of Toronto, Toronto, Ontario M5S3E1, Canada; 2 Department of Biological Chemistry and Molecular Pharmacology, Harvard Medical School and Center for Cancer Systems Biology, Dana-Farber Cancer Institute, Boston, Massachusetts 02115, USA

## 

One of the most surprising aspects of the completed human and mouse genome sequences [1-3] has been the relatively small number of protein-coding genes. The current estimate of <24,000 protein-coding genes in human and mouse is only four times that of budding yeast [4]. A complete encyclopedia of biochemical, cellular, and physiological gene functions is now an immediate rather than a long-term goal.

The unifying theme for papers in this supplement to *Genome Biology *is the automated inference of molecular function of gene products, and of their membership within cellular components and biological processes. In each study, thousands of variables describing genes and gene-gene relationships have been integrated using machine learning methods to infer Gene Ontology (GO) terms for essentially all genes in the studied genome.

Systematic biochemical and genetic experimentation in simpler model organisms has contributed to the rapid increase in the proportion of characterized genes. For example, about 80% of yeast genes have some annotated function [5]. Beyond simpler model systems such as yeast, however, cost and time requirements may preclude many systematic analyses, for example, resource-intensive phenotype assays in adult animals. Fortunately, efforts in simpler model organisms have illustrated that gene functions can be inferred on the basis of data types that are easier to collect systematically. Protein sequence features, expression patterns, and protein-protein interactions, for example, can provide powerful clues to function. This raises the prospect of directing resource-intensive experimentation toward the genes most likely to yield positive results. In yeast, this concept is well established, and the tradeoffs between performance measures, the efficacy of combinations of different data types in making different types of predictions, and the applicability of diverse inference algorithms are topics of active research.

Large scale experimentation in mammals is coming of age. A wide variety of mRNA expression analysis experiments are available in public data repositories, for example, the Gene Expression Omnibus [6]. The majority of human genes have at least one moveable open reading frame clone [7-9], enabling expression studies *in vitro *and in model systems. 'Knockdown' reagents targeting most mouse and human genes are now available [10], facilitating analysis of biochemical and cellular gene functions. Efforts are underway to create a mutant allele of each mouse gene [11], which will enable analysis of physiological and developmental roles.

The first paper in this supplemental issue [12] describes the 'MouseFunc' challenge, in which nine bioinformatics teams independently predicted mouse GO terms. Importantly, each used a common collection of training data and common benchmarks, which allowed comparison among the inference methods, data sets, and categories of gene functions. Predictions were tested using cross-validation (annotation for a subset of genes was hidden from the participants). Predictions were further tested by two forms of prospective evaluation: first, using GO annotations that had been added to the database since the inception of the study; and second, literature related to top-scoring novel predictions was investigated intensively by experienced mouse biologists.

Each of the companion papers in this issue is connected to the MouseFunc challenge, either in the nature of the algorithms used, in the datasets employed, or both. Guan and coworkers (led by Olga Troyanska) apply a support vector machine approach to predict mouse gene function [13]. They go on to apply this approach to the more tractable model eukaryote *Saccharomyces cerevisiae *and to test specific predictions experimentally. Mostafavi and colleagues (led by Quaid Morris) apply a ridge regression approach to predict mouse and yeast gene function [14]. The approach is quite fast, permitting their 'GeneMania' software to perform predictions 'on the fly' with a training set provided by the user. Kim and coworkers (led by Edward Marcotte) infer mouse gene function both directly and via a functional linkage network [15]. Functional linkage graphs contain connections between genes weighted by confidence that are functionally related [16]. Obozinski and colleagues (led by William Noble) investigate the possibility of inconsistency between predictions of different functions [17]. For example, it is possible for some approaches to assign a higher prediction score to 'DNA helicase activity' than to its logical parent term 'helicase activity'. They show that 'reconciliation' methods that enforce consistency between different GO term predictions can improve performance. Tian and coworkers [18] and Tasan and colleagues [19], both teams led by one of us (FPR), each combine guilt-by-profiling and guilt-by-association approaches to make predictions. Tian and coworkers describe the methodology and apply it to predict *S. cerevisiae *gene functions, while Tasan and colleagues apply the methodology to predict both functions and phenotypes for mouse genes.

Many other quantitative fields have benefitted by standardization of training and test sets. For example, the Critical Assessment of Techniques for Protein Structure Prediction (CASP) challenge [20] has made rigorous comparisons among protein structure predictions. This special issue suggests the value of similar standardization in the arena of function prediction.

Importantly, inferences about function and phenotype made in this issue are not black or white, but rather are expressed in shades of gray. Biology will long remain in the 'working model' phase, in which each statement about a gene's role must be accompanied by some uncertainty. An honest assessment of our uncertainties could allow us to direct resources efficiently to those experiments most likely to resolve these uncertainties. Quantitative predictions allow individual users requiring highly stringent predictions to impose a high prediction score threshold, while users may lower their threshold and include additional false positives if they wish to cast a wide net and catch more true positives.

The approaches taken in this issue have common limitations. To reduce the scope of the computational problem and eliminate the potential for inflated performance estimates due to circular reasoning, participants did not have access to GO annotations from other species. Although the training data did incorporate many previous transfers of annotation from other species by orthology, these methods could also benefit from a similar standardization and benchmarking strategy.

We also note that identifying the best strategies does not always help us to understand why the best strategies worked well. Because of the computationally intensive nature of function prediction, only a limited number of variant approaches were evaluated. A full factorial analysis of variations on the most successful strategies will help provide this understanding and allow future optimization.

The high precision of top predictions for many GO terms illustrates the richness and value of data sources that have become available for mammals over recent years. However, one lesson learned is that it is difficult to achieve both high precision and high recall. Currently, no algorithms achieve both for most functional categories. Improvements in either the inference methods, the problem setup, or in the information content of the data sets themselves will be needed in order to make a major dent in the more than 10,000 currently uncharacterized mouse and human genes.

## Abbreviations

GO, Gene Ontology.

## Competing interests

The authors declare that they have no competing interests.
